# A visual analysis of research hotspots and trends on macrodactyly between 2005 and 2024

**DOI:** 10.1186/s13023-025-04011-9

**Published:** 2025-09-24

**Authors:** Yuan Liu, Zong-You Yang, Chao-Jian Pang, Xiao-Bo Fan, Chen-Yang Zhao, Zhi-Kun Wei

**Affiliations:** 1 Department of Orthopaedic Surgery, the First Hospital of Handan, No. 25 Congtai Road, Handan, 050000 Hebei Province China; 2 Department of Orthopaedic Surgery, Hebei Medical University Third Hospital, Shijiazhuang, 050000 Hebei Province China

**Keywords:** Macrodactyly, Overgrowth, Management, Visualized, Hotspot

## Abstract

**Purpose:**

To conduct a visualization analysis of macrodactyly research from 2005 to 2024, providing a comprehensive overview of research trends, key contributors, and emerging topics.

**Methods:**

A visual analysis of macrodactyly related publications from 2005 to 2024 was conducted using the Web of Science Core Collection database. Publication trends, country and institutional contributions, author collaboration networks, and keyword co-occurrences were analyzed. Statistical analysis and visualization were performed using Microsoft Excel, R, VOSviewer, and CiteSpace.

**Results:**

One hundred and fifty-three publications were included. Annual publication trends showed fluctuations but an overall growth in interest over time, with notable growth from 2011 to 2014 peaking at 15 publications in 2014. The United States led with 128 publications, followed by China (60), Italy (40), Japan (36), and Turkey (27), with prominent institutions such as Mayo Clinic and Harvard University playing pivotal roles. Key authors like Dr. Marybeth Ezaki, working with the team at Texas Scottish Rite Hospital for Children, made substantial contributions to establishing diagnostic frameworks. Most importantly, keyword analysis revealed a fundamental shift in research focus from clinical and surgical themes (represented by keywords such as “foot,” “hand,” and “osteotomy” in early periods) to molecular and genetic investigations (characterized by “PIK3CA,” “activating mutations,” and “overgrowth” in recent years). The strongest citation burst was “overgrowth” (2016–2020), followed by genetic-related terms, with “activating mutations” representing the most recent trend (2020–2024), indicating increasing emphasis on PIK3CA mutations as the current research hotspot.

**Conclusion:**

This study highlights the evolution of macrodactyly research and reveals fluctuating publication trends and substantial contributions from key countries, authors, and institutions. The transition from clinical and surgical approaches to molecular and genetic investigations underscores advancements in the field. Future research should prioritize integrating genetic findings with clinical applications and advancing diagnostics and treatment strategies.

## Introduction

Macrodactyly is a rare congenital disorder characterized by abnormal overgrowth of fingers or toes. This condition invariably leads to both anatomical and functional deficiencies in the affected individuals [1]. Macrodactyly is believed to arise from gene mutations that stimulate cell growth and differentiation pathways [2, 3]. Recent molecular genetics studies have identified PIK3CA mutations as a central mechanism in macrodactyly pathogenesis [4, 5]. 

Although macrodactyly is uncommon, increasing case reports and research efforts over the past decades have notably enhanced our understanding of its etiology, clinical presentation, and treatment options [6–10]. However, existing research often focuses on isolated aspects of macrodactyly, such as surgical techniques, genetic mechanisms, or histopathological features, without providing a visualized and comprehensive analysis of the global research landscape [11–14]. This fragmentation is particularly problematic for rare diseases like macrodactyly, where research efforts are scattered across different institutions and countries, and the low prevalence results in limited case studies and fragmented literature [15]. Consequently, our understanding of the overall development and trends of macrodactyly research remains limited. A visualized approach to mapping the evolution of the scientific literature in this field is essential to uncover research gaps, identify influential studies, and facilitate future investigations.

Visualized analysis is a powerful method for evaluating scientific publications. Presenting publication patterns, citation networks, and collaborative trends through visualized figures and tables enables researchers to gain insights into the development of a field over time, identify research hotspots, and predict emerging trends more easily and comprehensively [16]. For rare disease research, visualized analysis enables tracking of how scientific understanding evolves over time [17]. Additionally, by mapping citation networks and collaborative patterns, this approach identifies key researchers and facilitates international cooperation, essential for pooling limited resources and patient cohorts [18]. Such an analysis is particularly valuable for rare disorders such as macrodactyly.

This study aimed to provide insights into the evolution of macrodactyly research, highlight key contributors and influential studies, and identify major research hotspots and emerging trends through a visualized analysis of publications from 2005 to 2024. Additionally, it seeks to promote greater awareness of macrodactyly among clinicians and researchers, fostering advancements in diagnosis, management, and treatment. Through this analysis, this study contributes to a more integrated and forward-looking understanding of macrodactyly.

## Methods

### Data sources and search strategy

We retrieved relevant literature from the Web of Science Core Collection (WOSCC) database. Our keyword selection strategy was based on established medical terminology and synonymous terms commonly used in the literature to describe macrodactyly. “Macrodactyly” is the standard medical term as defined in contemporary orthopedic and plastic surgery literature. “Megalodactyly” is a historically used synonym that appears in earlier publications. “Digital gigantism” and “giant digit” are descriptive terms that reflect the clinical presentation of the condition. The search strategy was as follows: (((TS=(macrodactyly)) OR TS=(Megalodactyly)) OR TS=(Digital gigantism)) OR TS=(Giant digit)). We included studies published between January 2005 and July 2024 that were written exclusively in English. Publication type was limited to articles and reviews. Manual screening was performed to exclude irrelevant publications. Manual screening was performed to exclude irrelevant publications based on the following criteria: (1) studies in which macrodactyly-related terms appeared in the topic fields but the main focus of the article was not macrodactyly; (2) editorial material, letter, proceeding paper, book chapters, early access, meeting abstract; (3) duplicate publications; (4) publications where full text or abstract was unavailable for assessment. All the data collected in this study were publicly available, and ethical approval was not required.

### Data collection

We downloaded and retrieved basic information for all identified publications, including titles, keywords, abstracts, publication years, authors, impact factors, H-index, countries or regions, and affiliations. Duplicate checking was performed using the identifiers including DOI, title, and author information. When duplicate publications were identified, only the most complete version was retained for analysis. The data search and collection were conducted independently by two researchers and verified by a third researcher to ensure accuracy.

### Statistical analysis and visualization

We used Microsoft Excel 2021 (Microsoft Corp., Redmond, WA, USA), bibliometrix package in R (Version 4.3.2), VOSviewer (Version 1.6.20), and CiteSpace (Version 6.2. R4) for data analysis and visualization [19, 20]. Microsoft Excel was used to record and analyze the data, including the creation of graphs depicting the number of publications per year. The bibliometric online analysis platform was used to visualize the distribution of publications by country, where darker colors indicate higher research outputs.

VOSviewer facilitates the visual analysis of co-authorship and keyword co-occurrences. Specifically, nodes on the maps represent various elements such as countries/regions, authors, and keywords. Larger node sizes indicate higher frequencies of publications, citations, or occurrences, whereas the links between elements represent the strength of associations. Total link strength (TLS) was used to determine the strength of these connections.

CiteSpace was employed to analyze the burst keywords. To identify emerging research trends, keyword bursts were analyzed sequentially, and the top 10 keywords with the strongest citation bursts were selected for a detailed analysis.

## Results

### Publication trends

**Fig. 1:**
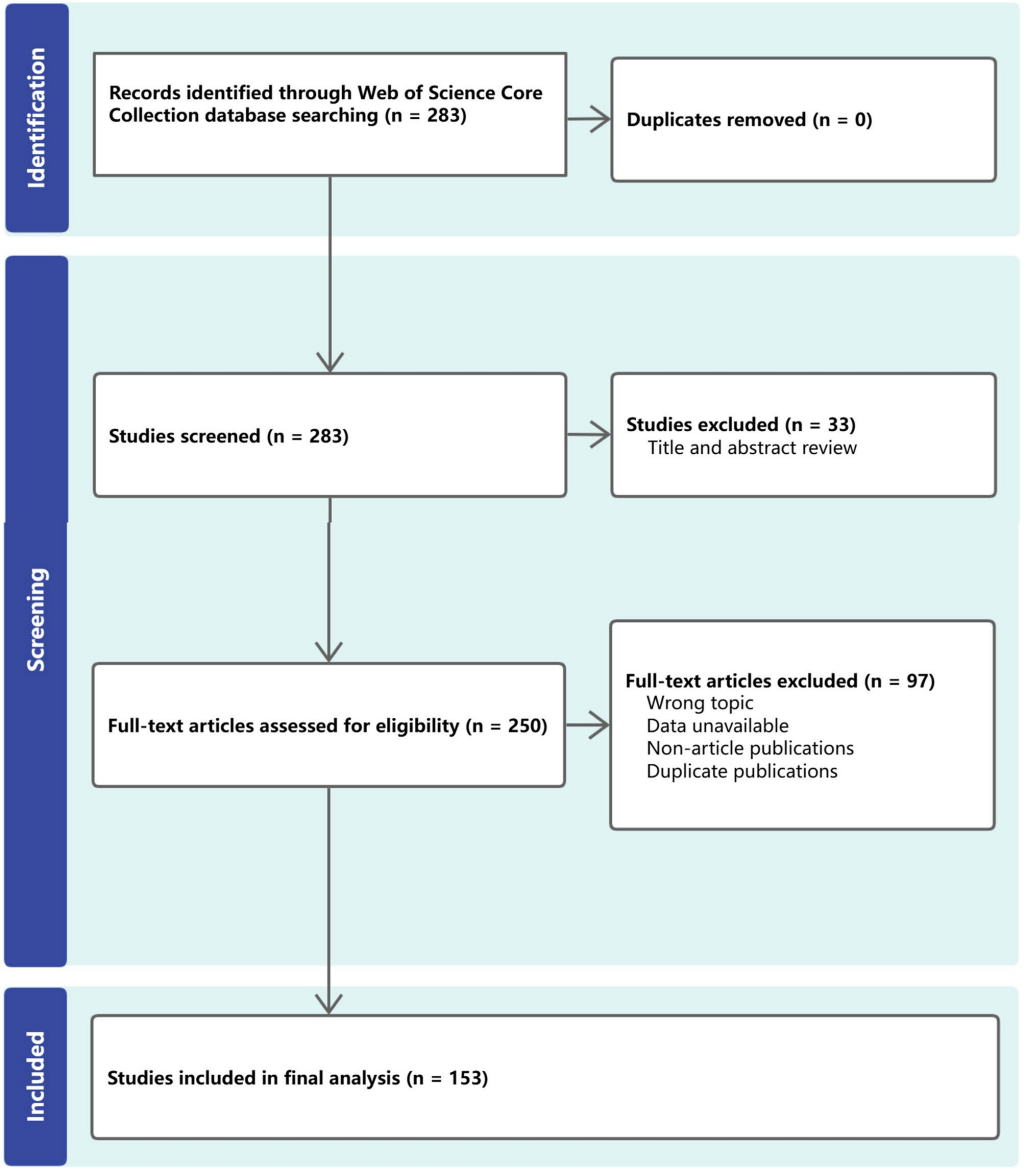
Flowchart of literature selection process. Initial search yielded 283 publications from Web of Science Core Collection. First screening excluded 33 non-article publications by type. Second screening excluded 97 articles based on the following criteria: (1) publications not directly related to macrodactyly; (2) conference abstracts, editorial materials, letters, and corrections that were initially misclassified as articles; (3) duplicate publications; (4) publications where full text or abstract was unavailable for assessment. Final analysis included 153 articles meeting all inclusion criteria

**Fig. 2:**
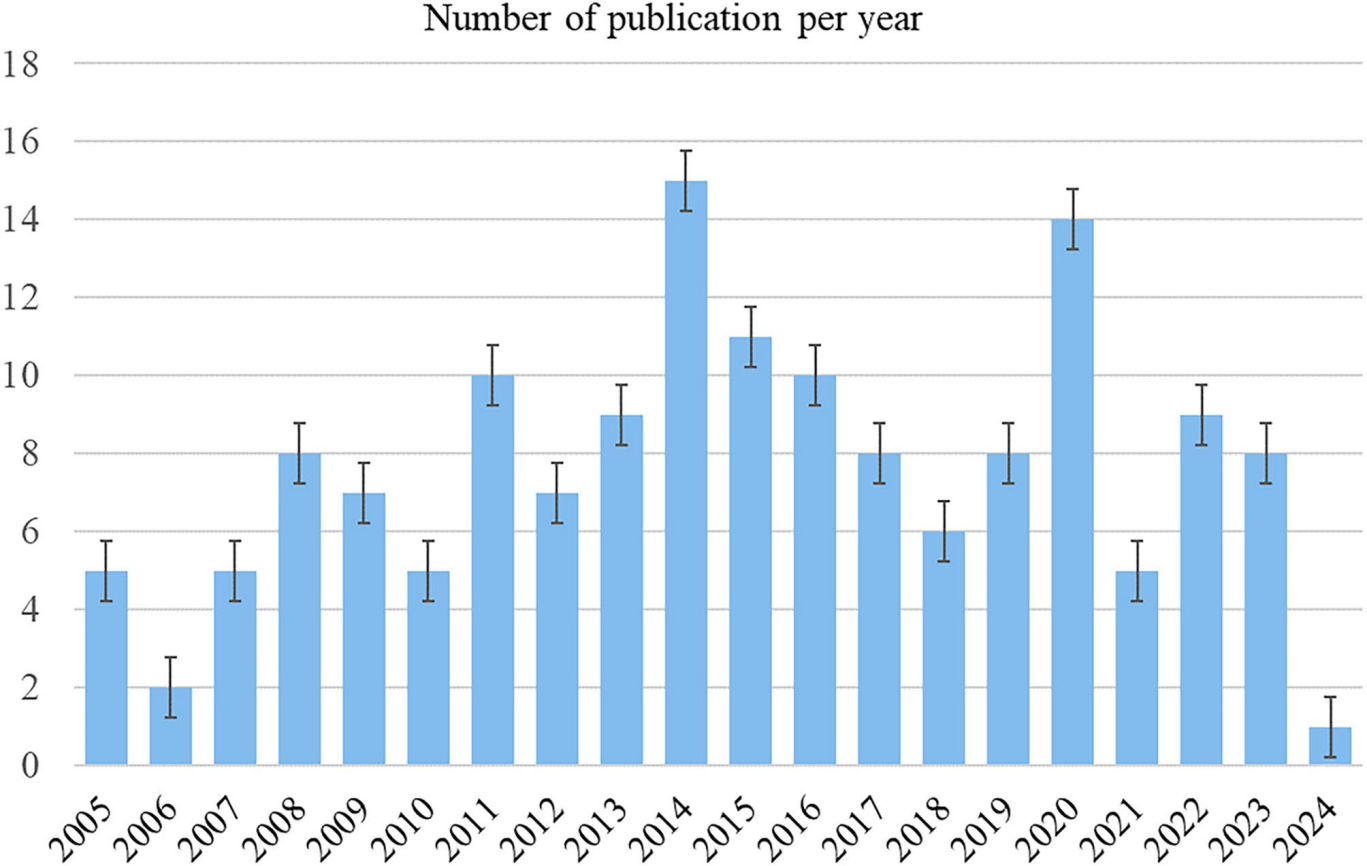
Bar graph of publication number every year from 2005 to 2024

As shown in Figs. 1 and 283 publications were collected between 2005 and 2024, and 153 articles were ultimately included in the study after excluding unrelated publications. The analysis of annual publications from 2005 to 2024 revealed a fluctuating trend, reflecting the rarity of macrodactyly. Between 2005 and 2010, the number of publications varied between two and eight per year. Notable growth occurred from 2011 to 2014, peaking at 15 publications in 2014. From 2015 to the present, the annual output has remained unstable, fluctuating around nine publications per year (Fig. 2).

### Distribution among countries or regions

**Fig. 3:**
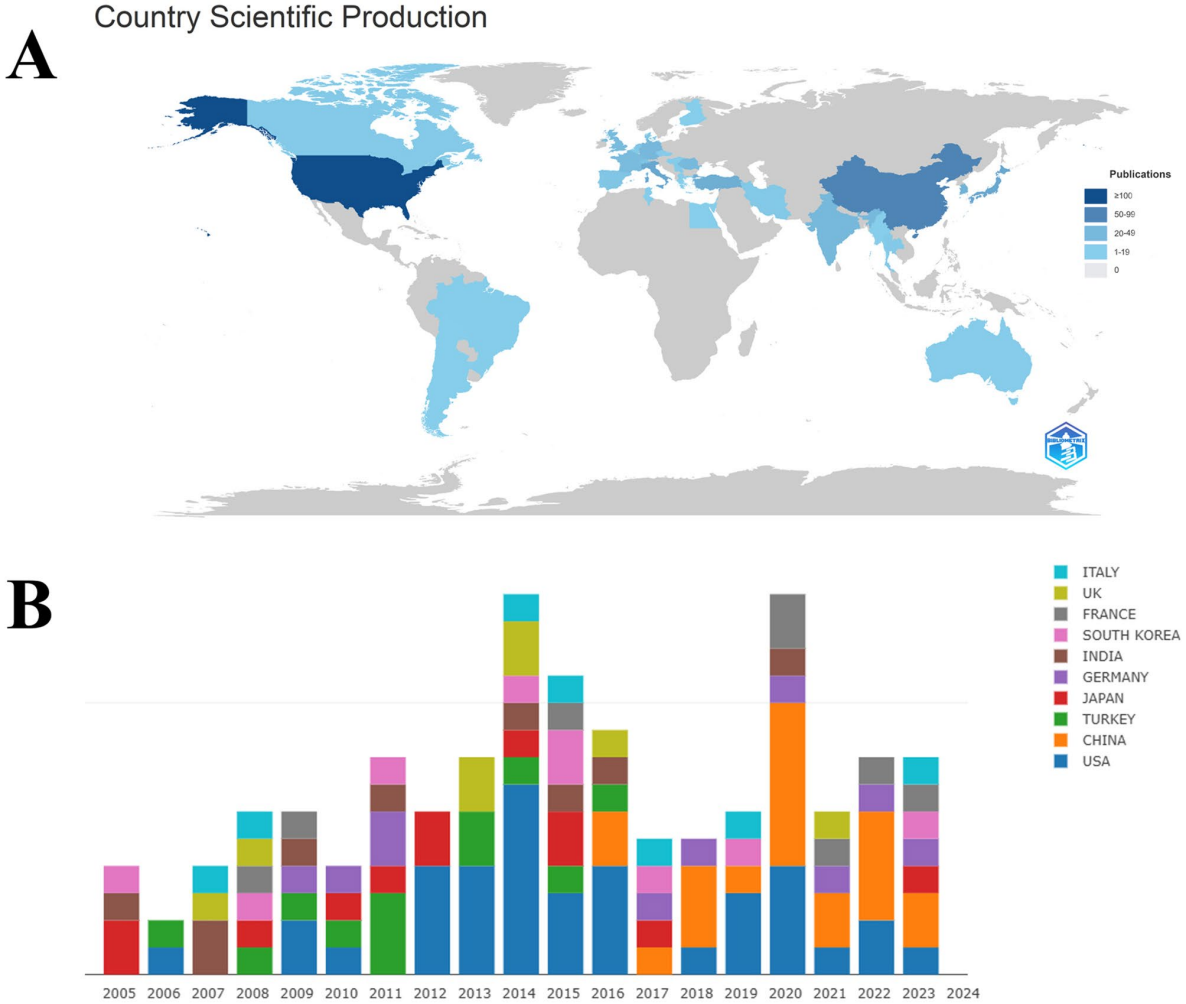
The global contributions to macrodactyly research. (**A**) The world map displays the distribution of research. The density of colors indicates the outputs volume. (**B**) The bar graph lists the top 10 productive countries in macrodactyly research

A total of 41 countries or regions have published research on macrodactyly. The distribution map highlights concentrated research activity in North America, Europe, and Asia, with notable contributions from South America and Australia (Fig. 3A). The United States has 128 publications, underscoring its dominant role in macrodactyly research. China has 60 publications, while Italy (40), Japan (36), and Turkey (27) have also demonstrated considerable outputs. The top ten high-output countries from 2005 to 2024 are shown in Fig. 3B. The United States leads the number of publications annually, peaking in 2011 and 2015. The number of publications in China increased sharply in 2014. Italy, Japan, and Turkey have made steady contributions over the years.

### Analysis of authors

**Fig. 4:**
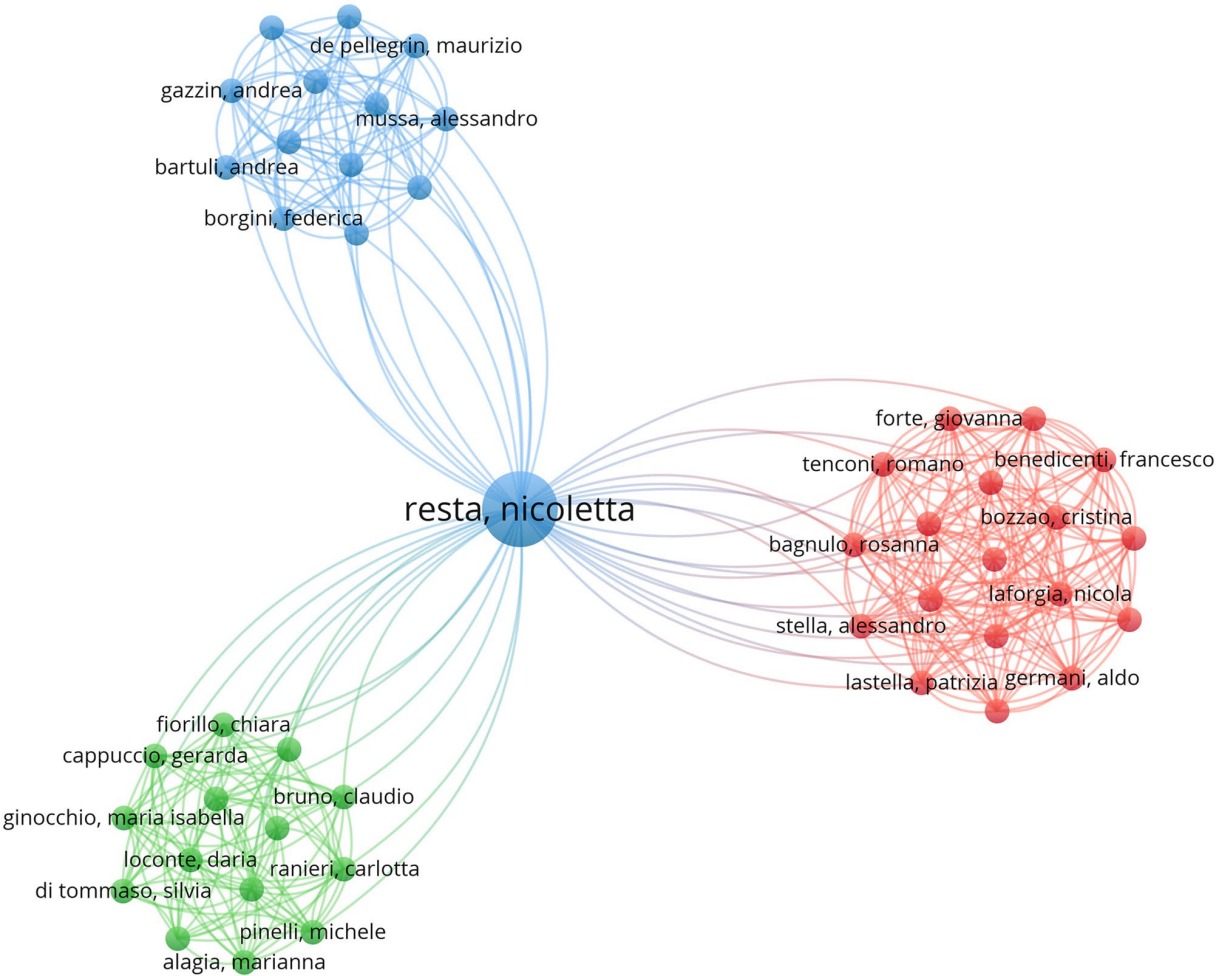
Network visualization map of co-author analysis. Each node represents a different author, and the connecting lines represent collaborative relationships. Colors represent different collaboration clusters identified through network analysis. The central position of Nicoletta Resta demonstrates her role as a key connector between multiple research groups. The network structure reveals both tightly connected research teams and bridge authors who facilitate inter-group collaborations

From 2005 to 2024, 736 authors participated in the research on macrodactyly. The top ten authors by output are listed in Table 1. Robert J. Spinner stands out, with eight publications and 148 citations. He also had the highest H-index of seven, demonstrating a strong influence on the research community. Mark A. Mahan followed 6 publications and 78 citations. Marybeth Ezaki, with five publications and 500 citations, has an average citation per item (ACI) of 100.4. Her most cited article is a review of the PIK3CA-Related Overgrowth Spectrum [4]. Notably, Nicoletta Resta, with 3 publications and 64 citations, showed a strong total link strength (TLS) of 45. As Fig. 4 shows, Nicoletta Resta’s highest TLS highlights her extensive collaboration in macrodactyly research. Kohji Miura and Keiichi Ozono, with three publications and 168 citations, also stand out. Other notable authors include Gang Han, Bin Wang, and Shengbo Zhou, each with 4 publications and 26 citations. Kimberly K. Amrami has 4 publications and 81 citations. The top 10 most cited articles in macrodactyly research are presented in Table 2. The most cited article is “Clinical delineation and natural history of the PIK3CA-related overgrowth spectrum” with 217 citations, followed by “Somatic gain-of-function mutations in PIK3CA in patients with macrodactyly” with 126 citations. The third most cited work, “Characterization of a distinct syndrome that associates complex truncal overgrowth, vascular, and acral anomalies: a descriptive study of 18 cases of CLOVES syndrome” (117 citations), expanded the understanding of overgrowth syndromes beyond isolated macrodactyly.

**Table 1:** The top 10 output authors in macrodactyly researches

Rank	Author	Counts	Citation counts	TLS	H-index	ACI
1	Robert J. Spinner	8	148	28	7	18.50
2	Mark A. Mahan	6	78	18	5	13.00
3	Marybeth Ezaki	5	500	26	5	100.40
4	Gang Han	4	26	26	3	6.75
5	Bin Wang	4	26	26	3	6.75
6	Shengbo Zhou	4	26	26	3	6.75
7	Kimberly K. Amrami	4	81	13	4	20.25
8	Nicoletta Resta	3	64	45	2	21.33
9	Kohji Miura	3	168	33	3	56.00
10	Keiichi Ozono	3	168	33	3	56.00

**Table 2:** The top 10 cited articles in macrodactyly researches

Rank	Title	Total citation
1	Clinical delineation and natural history of the PIK3CA-related overgrowth spectrum [30]	217
2	Somatic gain-of-function mutations in PIK3CA in patients with macrodactyly [31]	126
3	Characterization of a distinct syndrome that associates complex truncal overgrowth, vascular, and acral anomalies: a descriptive study of 18 cases of CLOVES syndrome [34]	117
4	An Overgrowth Disorder Associated with Excessive Production of cGMP Due to a Gain-of-Function Mutation of the Natriuretic Peptide Receptor 2 Gene [35]	94
5	PIK3CA Activating Mutations in Facial Infiltrating Lipomatosis [28]	72
6	Overgrowth Syndrome Associated With a Gain-of-Function Mutation of the Natriuretic Peptide Receptor 2 (NPR2) Gene [36]	66
7	Molecular and Functional Characterization of Three Different Postzygotic Mutations in PIK3CA-Related Overgrowth Spectrum (PROS) Patients: Effects on PI3K/AKT/mTOR Signaling and Sensitivity to PIK3 Inhibitors [3]	65
8	Lipofibromatous Hamartoma of the Median Nerve: A Comprehensive Review and Systematic Approach to Evaluation, Diagnosis, and Treatment [37]	56
9	Presentation and Treatment of Macrodactyly in Children [1]	36
10	Somatic PIK3R1 variation as a cause of vascular malformations and overgrowth [38]	33

### Analysis of institutions

**Fig. 5:**
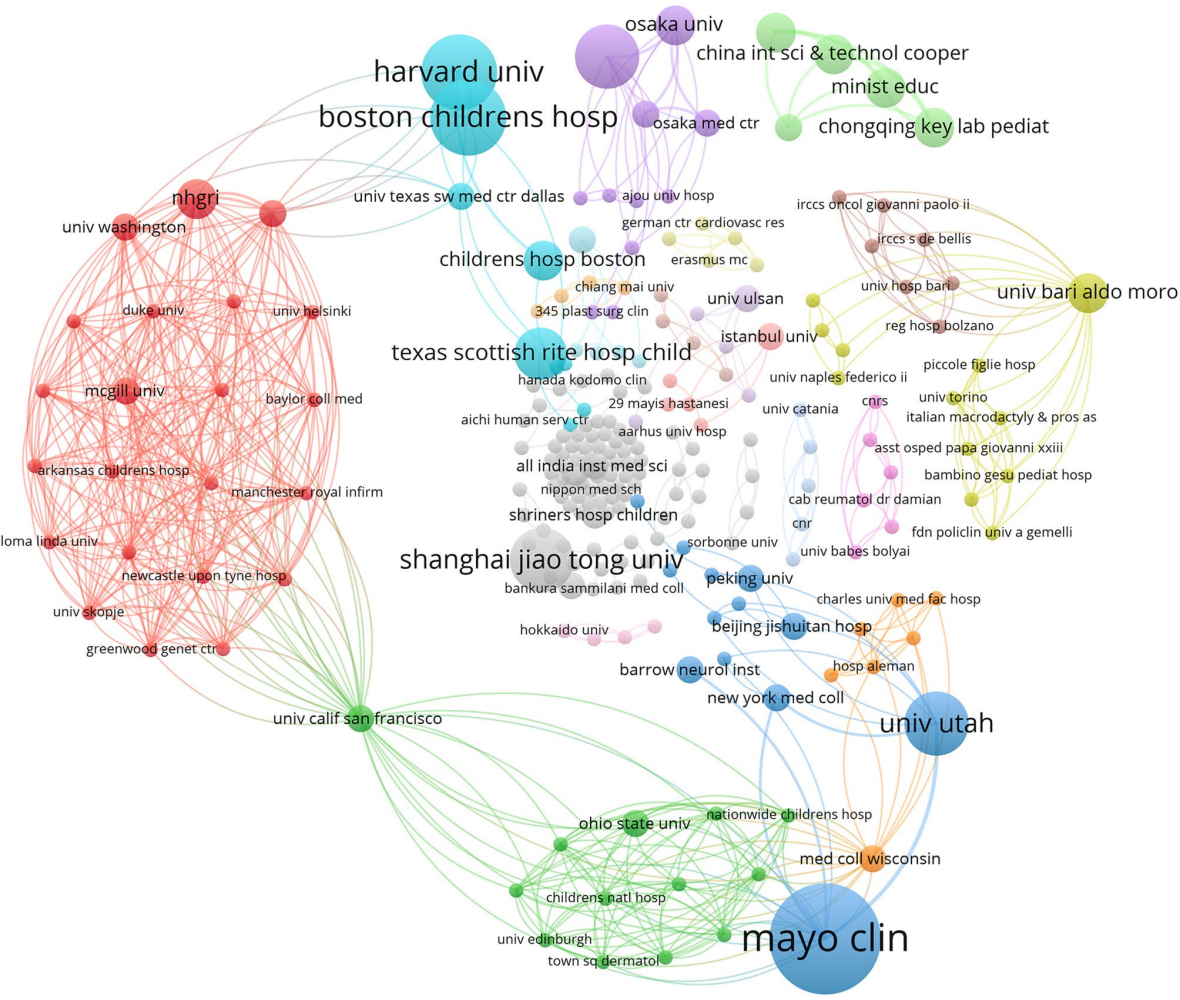
The network map of the institutions. The size of the nodes indicates the volume of outputs. The connecting lines represent collaborative relationships

**Fig. 6:**
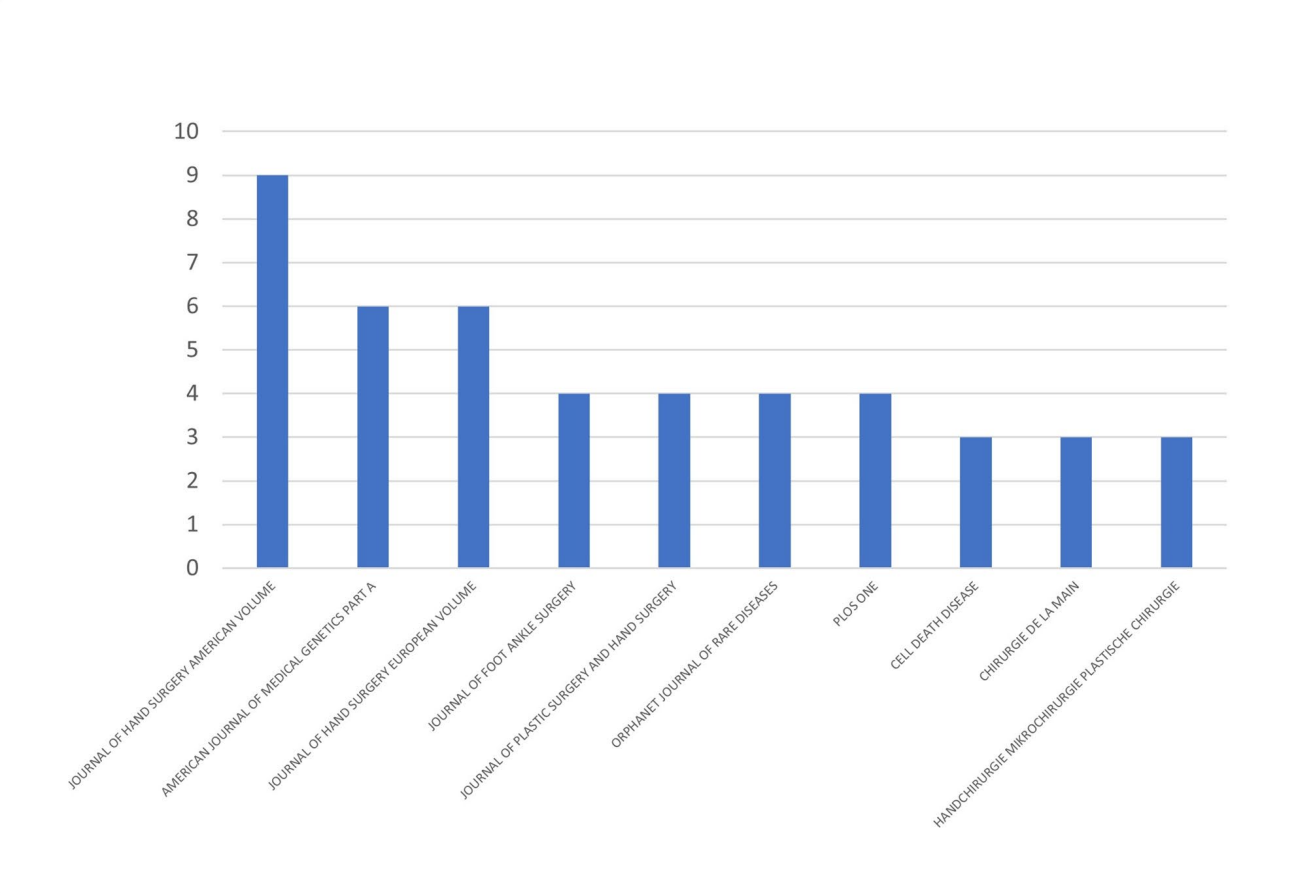
Distribution of macrodactyly research publications across the top 10 journals

A total of 284 institutions contributed to research on macrodactyly. A network visualization of the institutional analysis is shown in Fig. 5. The size of each circle indicates the number of publications, whereas the lines between institutions represent collaboration. The Mayo Clinic led with nine publications, followed by Harvard University and Boston Children’s Hospital, each with six publications. Other notable institutions included the University of Utah (five publications), Seoul National University (five publications), and Shanghai Jiao Tong University (five publications). The University of California, San Francisco (UCSF) has the highest TLS of 36, indicating high-frequency collaboration with other institutions. The NHGRI, the University of Cambridge, and the University of Washington have a TLS of 27, illustrating their collaborative efforts in macrodactyly research. The distribution of macrodactyly research across journals is presented in Fig. 6. The Journal of Hand Surgery American Volume leads with 9 publications, followed by the American Journal of Medical Genetics Part A with 6 publications, and the Journal of Hand Surgery European Volume with 6 publications.

### Analysis of keywords and hotspots

**Fig. 7:**
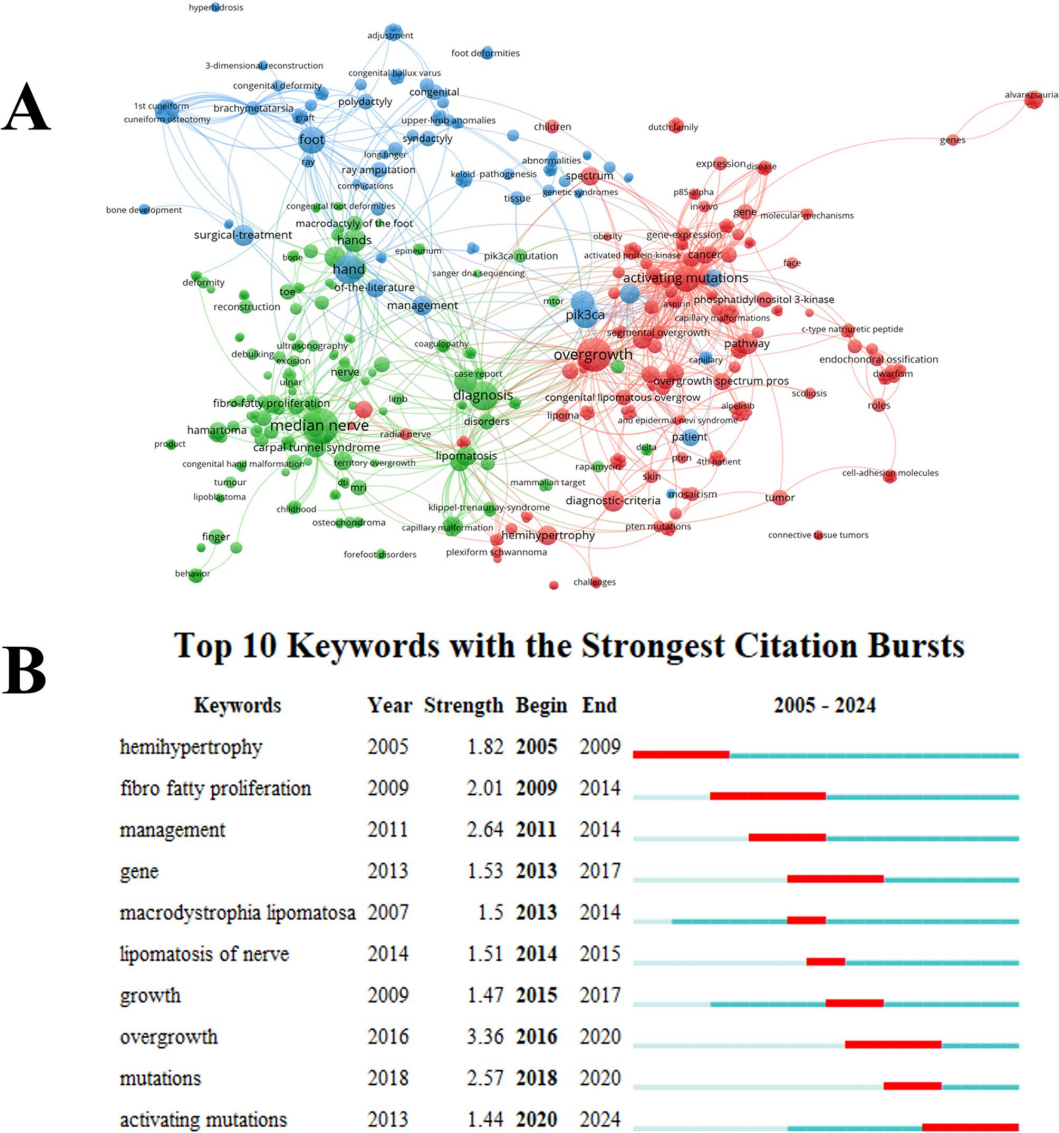
The Co-occurrence analysis of global research on macrodactyly. (**A**) Mapping of keywords in the research. Each node represents a keyword, and the connections illustrate co-occurrence relationships. (**B**) The top 10 keywords with the strongest citation bursts from 2005 to 2024. Citation bursts were calculated using the burst detection algorithm in CiteSpace, which identifies keywords that have experienced a sharp increase in citation frequency over a specific time period. The strength value reflects the intensity of the burst, while the begin and end years indicate the duration during which the keyword was cited considerably more often than in other periods. The blue line indicates the time interval, while the red line represents the duration of each keyword’s strongest citation burst

A total of 603 keywords were retrieved from all included publications. Network visualization, as shown in Fig. 7A, illustrates the co-occurrence analysis of the keywords. These keywords are divided into three main research clusters, each represented by a different color. The blue cluster focuses on clinical features and surgical treatment of macrodactyly, using keywords such as “foot,” “polydactyly,” and “osteotomy.” The green cluster reflects research related to diagnosis and syndromes, featuring keywords like “median nerve,” “ultrasonography,” and “MRI.” The red cluster emphasizes the molecular mechanisms and genetics, including keywords such as “PIK3CA,” “activating mutations,” and “overgrowth.” Based on the keyword co-occurrence network, “foot” and “hand” are the most prominent nodes, indicating that macrodactyly primarily affects these two areas. The close association of “overgrowth,” “PIK3CA,” and “activating mutations” underscores the importance of molecular mechanism studies in macrodactyly.

The top ten keywords with the strongest citation bursts from 2005 to 2024 are presented in Fig. 7B. The strongest citation burst was “overgrowth” (strength 3.36) from 2016 to 2020, highlighting its central role in recent research. “Management” received growing interest from 2011 to 2014. Genetic studies began to gain focus with keywords such as “gene” (2013–2017) and “mutations” (2018–2020). The most recent trend is “activating mutations” (2020–2024), indicating the current emphasis on specific genetic mechanisms.

## Discussion

This visual analysis provides an integrated and forward-looking understanding of macrodactyly. By analyzing publication trends, global research contributions, key authors and institutions, and research hotspots, our study highlights both the progress made and the persistent challenges in understanding and managing this rare disorder.

Publication trends in macrodactyly research over the last two decades have fluctuated. Similar to other rare diseases, this variability is often due to the small sample sizes and limited resources [21–23]. From 2005 to 2010, publication numbers remained low and stable, reflecting the early stages of macrodactyly research. The dramatic growth observed from 2011 to 2014, peaking at 15 publications in 2014. Although the number of publications declined slightly after 2014, the average annual volume remained higher than the pre-2014 level. This trend suggests that more attention has been directed towards this topic. This fluctuating pattern highlights the need for enhanced international collaboration to pool resources and share expertise in rare disease research.

The global distribution of research identifies the United States as the dominant leader with 128 publications. This leadership aligns with its advanced investment and research infrastructure for rare-disease studies. Contributions from countries such as China, Italy, Japan, and Turkey further demonstrate a growing international interest in macrodactyly [2, 11, 24–26]. In particular, the marked increase in publications from China since 2014 may correspond not only to improved research capacity but also to broader access to advanced genetic diagnostic technologies, which have been pivotal in redefining macrodactyly within the framework of PIK3CA-related overgrowth spectrum (PROS). Rather than merely representing output volume, this international expansion of research activity plays a critical role in generating diverse clinical and molecular data. Such contributions are essential for clarifying phenotypic variability, validating genotype–phenotype correlations across populations, and ultimately facilitating more personalized approaches to diagnosis and management. The analysis of citation reveals the transformative impact of genetic discoveries on macrodactyly research. The two most cited articles by Keppler-Noreuil et al. and Rios et al. fundamentally shifted the field from descriptive clinical studies to mechanistic investigations. The high citation count of the PIK3CA-related overgrowth spectrum paper reflects its role in establishing diagnostic criteria.

Strong total link strength (TLS) among authors is a positive indicator for macrodactyly research [20, 27]. Such collaborations are essential for addressing the challenges posed by the limited cases and resources associated with rare disease studies. Marybeth Ezaki stands out for her high citation counts and long-standing contributions to the clinical understanding of macrodactyly, particularly through her co-authorship of seminal papers in the field. One of the most influential is the study by Keppler-Noreuil et al. (2015) [4]which she co-authored, proposing a standardized diagnostic framework for the PIK3CA-related overgrowth spectrum. This work was pivotal in shifting macrodactyly from being viewed as an isolated morphological anomaly to being understood as part of a broader spectrum of somatic mosaic disorders. The paper outlined precise clinical criteria, differential diagnoses, and indications for genetic testing. This publication not only provided clarity in clinical diagnosis but also formed a foundational reference for subsequent genetic and mechanistic studies, helping to unify the language and direction of research efforts globally.

The institutional landscape of macrodactyly research reveals a complex network of specialized medical centers that reflects the technical demands of treating this rare condition. The prominence of institutions such as the Mayo Clinic, Harvard University, and Boston Children’s Hospital in macrodactyly research aligns with their reputation as leading centers for healthcare. The strong collaborative links between institutions underscore the value of multi-center research in advancing the field. The University of California, San Francisco’s (UCSF) highest total link strength reveals a distinctive collaborative model in rare disease research. UCSF’s strengths in genetic research and plastic surgery, making it an ideal partner for institutions seeking to combine molecular investigations with clinical expertise. This high TLS suggests that UCSF serves as a bridge between different types of institutions—academic research centers, clinical hospitals, and specialized surgical programs. These institutions’ contributions are particularly noteworthy as they represent different healthcare systems and patient populations, potentially offering diverse perspectives on treatment approaches. This journal distribution reflects the multidisciplinary nature of macrodactyly research, spanning specialized hand surgery journals, medical genetics publications, and general medical journals.

Keyword analysis has revealed a marked evolution in the research focus over the past two decades. Early research primarily emphasized clinical and surgical themes, with keywords such as “foot,” “hand,” and “osteotomy.” In contrast, recent years have witnessed a shift towards molecular and genetic investigations, with keywords such as “PIK3CA,” “activating mutations,” and “overgrowth” [28–30]. This shift aligns with important studies by Rios et al., who identified somatic PIK3CA mutations in macrodactyly patients [31]. This shift presents opportunities for more personalized patient care. Understanding the genetic basis of macrodactyly could enable earlier diagnosis through prenatal testing, allow for risk stratification based on mutation type, and potentially guide surgical timing and extent. The identification of PIK3CA mutations has already led to investigational use of PI3K inhibitors in related overgrowth syndromes, suggesting that similar approaches might benefit macrodactyly patients in the future. Moreover, genetic insights may help distinguish true macrodactyly from other overgrowth conditions, ensuring more accurate diagnosis and appropriate treatment planning.

The focus on genetic mechanisms is promising for advancing targeted therapies and developing more precise diagnostic methods. Considering these findings, future studies should focus on translating research outcomes into clinical applications. First, efforts should be directed towards developing targeted therapies to inhibit pathways associated with PIK3CA mutations. Second, researchers should explore the roles of other genes in macrodactyly. Third, multicenter studies should be conducted to validate the findings. Finally, greater efforts should be directed towards integrating genetic data with clinical, imaging, and pathological findings to enhance diagnostic tools and optimize treatment strategies.

Through comparative analysis with other rare diseases, we identified distinctive characteristics of macrodactyly research. Institutional collaboration networks show higher centralization around major medical centers (Mayo Clinic, Harvard) compared to the distributed networks typical of rare metabolic disorders [32]. This centralization reflects macrodactyly’s specialized surgical expertise requirements. Geographically, the US dominance (128 publications) is more pronounced than other rare skeletal disorders, with European contributions comprising only 25–30% compared to 40–45% in osteogenesis imperfecta research [33]. This approach not only highlights macrodactyly research uniqueness but also provides targeted guidance for future development.

However, this study had several limitations. Our study relied on the data retrieved from a single database (WOSCC), which may have excluded relevant publications from other sources such as PubMed or Scopus. This limitation could result in an incomplete representation of the global research landscape, especially contributions from countries where researchers primarily publish in local journals not indexed in WOSCC. Additionally, most of the included publications were in English and published after 2005, potentially introducing language and selection bias. Non-English literature, particularly from countries like China, Japan, and South Korea, may contain valuable clinical insights and treatment innovations that remain inaccessible to the international community. Future studies should consider incorporating multiple databases and multilingual publications to provide a more comprehensive analysis of this rare disease.

## Conclusion

This study provides valuable insights into macrodactyly research by highlighting key trends, global contributions, and research hotspots. Fluctuating publication trends reflect the challenges of studying rare disorders. The United States leads research output, followed by China and other notable contributors such as Italy, Japan, and Turkey. Key authors, such as Marybeth Ezaki, and prominent institutions, including the Mayo Clinic and Harvard University, play critical roles in advancing the field. Research hotspots have shifted from clinical and surgical approaches to molecular and genetic research. Future efforts should focus on integrating these findings to optimize diagnostics and treatment strategies.

## Data Availability

Data used to support the findings of this study are available from the corresponding author upon request.
